# The Polish Konik Horse: A Multidisciplinary Review of Its Origin, Genetics, Ecology, Health, Behaviour and Reproductive Biology

**DOI:** 10.3390/ani16172784

**Published:** 2026-09-04

**Authors:** Marta Siemieniuch-Tartanus

**Affiliations:** Department of Large Animal Diseases with Clinic, Warsaw University of Life Sciences, Nowoursynowska 100, 02-787 Warsaw, Poland; marta_siemieniuch-tartanus@sggw.edu.pl

**Keywords:** Polish Konik horse, native horse breed, conservation genetics, conservation grazing, environmental adaptation, reproductive biology, behaviour, welfare

## Abstract

The Polish Konik horse (PKH) is one of Europe’s best-known native conservation breeds. Traditionally associated with the extinct Eurasian tarpan and conservation grazing, the breed has recently become the subject of multidisciplinary research encompassing genetics, ecology, health, behaviour and reproduction. This narrative review summarises current knowledge on the biological characteristics and contemporary scientific significance of the PKH. Literature published between 2005 and 2026 was identified through searches of PubMed, Scopus, Web of Science and Google Scholar and narratively synthesised. Available evidence suggests that, despite severe historical bottlenecks, the PKH has retained considerable genetic diversity and its characteristic maternal and paternal founder-line structure. Recent molecular studies have revised traditional concepts of the breed’s origin, while ecological research supports its important role in conservation grazing and wetland restoration. Behavioural and reproductive studies indicate stable temperament, high reproductive efficiency and adaptation to extensive management systems. However, current knowledge is derived predominantly from observational studies, with relatively few comparative investigations and limited genomic and longitudinal data. The PKH represents a valuable model for research on conservation genetics, environmental adaptation, animal welfare, reproductive biology and ecosystem management. Further interdisciplinary studies are needed to strengthen the evidence base for conservation and breeding strategies.

## Simple Summary

The Polish Konik horse is one of the best-known native horse breeds in Europe and represents an important component of Polish and European biocultural heritage. Historically associated with the extinct Eurasian tarpan, the breed was initially regarded mainly as a primitive relic population maintained under semi-feral conditions. However, contemporary research has substantially broadened scientific understanding of the breed. Studies published over the last two decades have demonstrated that Polish Konik horses possess considerable genetic diversity despite historical bottlenecks and a limited founder population. At the same time, the breed gained increasing importance in conservation grazing, biodiversity protection and wetland management due to its environmental resilience and adaptation to extensive systems. Behavioural studies additionally showed relatively stable temperament, low emotional reactivity, good adaptation to semi-feral management, and adequate usability. Recent investigations concerning respiratory health, reproduction and microbiome composition further highlighted the breed’s value as a model for studying adaptation and welfare in low-input horse populations. This review summarises current knowledge on the origin, genetics, ecology, health, and behaviour of the Polish Konik horse and discusses its contemporary role in conservation-oriented livestock systems.

## 1. Introduction

Native livestock breeds constitute an important component of global animal genetic resources because they combine unique genetic diversity, environmental adaptation and cultural heritage developed under local production systems [1,2]. In recent years, increasing attention has been directed toward native horse breeds, which are regarded not only as reservoirs of genetic diversity but also as valuable models for studying adaptation to extensive management systems, biodiversity conservation and sustainable livestock production [3,4].

Among European native horse breeds, the Polish Konik horse (PKH, *Equus caballus*) occupies a unique position. The breed originated from conservation efforts initiated by Tadeusz Vetulani, who attempted to reconstruct a horse phenotypically resembling the extinct Eurasian tarpan (*Equus ferus ferus*). As a result, the PKH became closely associated with the long-standing hypothesis that it is the closest living descendant of the European wild horse [5,6,7]. Although this concept has profoundly influenced both scientific and public perception of the breed for decades, recent advances in molecular genetics and archaeogenomics have substantially revised our understanding of its origin [8,9,10,11,12,13,14].

Today, the PKH is recognised as one of the most important native horse breeds in Central Europe and represents an integral component of Polish biocultural heritage. Owing to its adaptation to low-input management systems and its hardiness and ability to thrive under semi-feral conditions, the breed has become increasingly used in conservation grazing programmes aimed at maintaining biodiversity and restoring semi-natural habitats [15,16,17,18]. The targeted breeding of Konik horses is a relatively recent phenomenon, as it essentially spans the past 100 years [5,11] and, from the outset, had different goals than the breeding and selection of sport horses [19]. At the same time, the Polish Konik has attracted growing scientific interest as a model for investigating conservation genetics, environmental adaptation, behaviour, welfare, reproduction and health in primitive horse populations [20,21,22,23,24,25,26,27,28,29,30,31,32]. The evolution of scientific perspectives on the PKH is presented in Table 1.

Despite the increasing number of publications published during the last two decades, the available literature remains highly fragmented. Previous studies have generally focused on individual aspects of the breed, including its origin, genetic diversity, ecological role, behaviour or veterinary characteristics, whereas comprehensive interdisciplinary syntheses are lacking. Moreover, much of the earlier literature portrayed the PKH primarily as a relic of the tarpan or as a conservation grazer, while more recent studies have considerably expanded this perspective by demonstrating the breed’s importance for research on animal welfare, reproductive biology, environmental adaptation, microbiome composition and sustainable management.

Therefore, this narrative review aimed to critically synthesise the scientific evidence published between January 2005 and May 2026 by addressing the following research questions:

(i) How has contemporary genetic and archaeogenomic research reshaped our understanding of the origin and ancestry of the PKH?
(ii) What is the current state of knowledge regarding the maternal and paternal genetic structure of the breed, and what are the implications for conservation breeding?
(iii) What evidence supports the ecological role of the PKH in conservation grazing, biodiversity maintenance and landscape management?
(iv) What is currently known about the health, reproductive biology and environmental adaptation of the breed under both semi-feral and managed conditions?
(v) What behavioural, welfare and utility traits characterise the PKH, and how do they contribute to its conservation and practical use? By integrating findings from genetics, ecology, veterinary medicine and animal science, this review aims to provide a comprehensive overview of the current state of knowledge and to identify future directions for research and conservation of this unique native horse breed.

## 2. Materials and Methods

### 2.1. Literature Search Strategy

A narrative review, supported by a systematic literature search, was conducted to identify scientific publications on the PKH published between 1 January 2005 and 31 May 2026. The review was prepared between March and May 2026 in accordance with the general recommendations for review articles published in Animals MDPI editorial guidelines, using four electronic databases: PubMed, Scopus, Web of Science Core Collection, and ScienceDirect.

**Figure 1:**
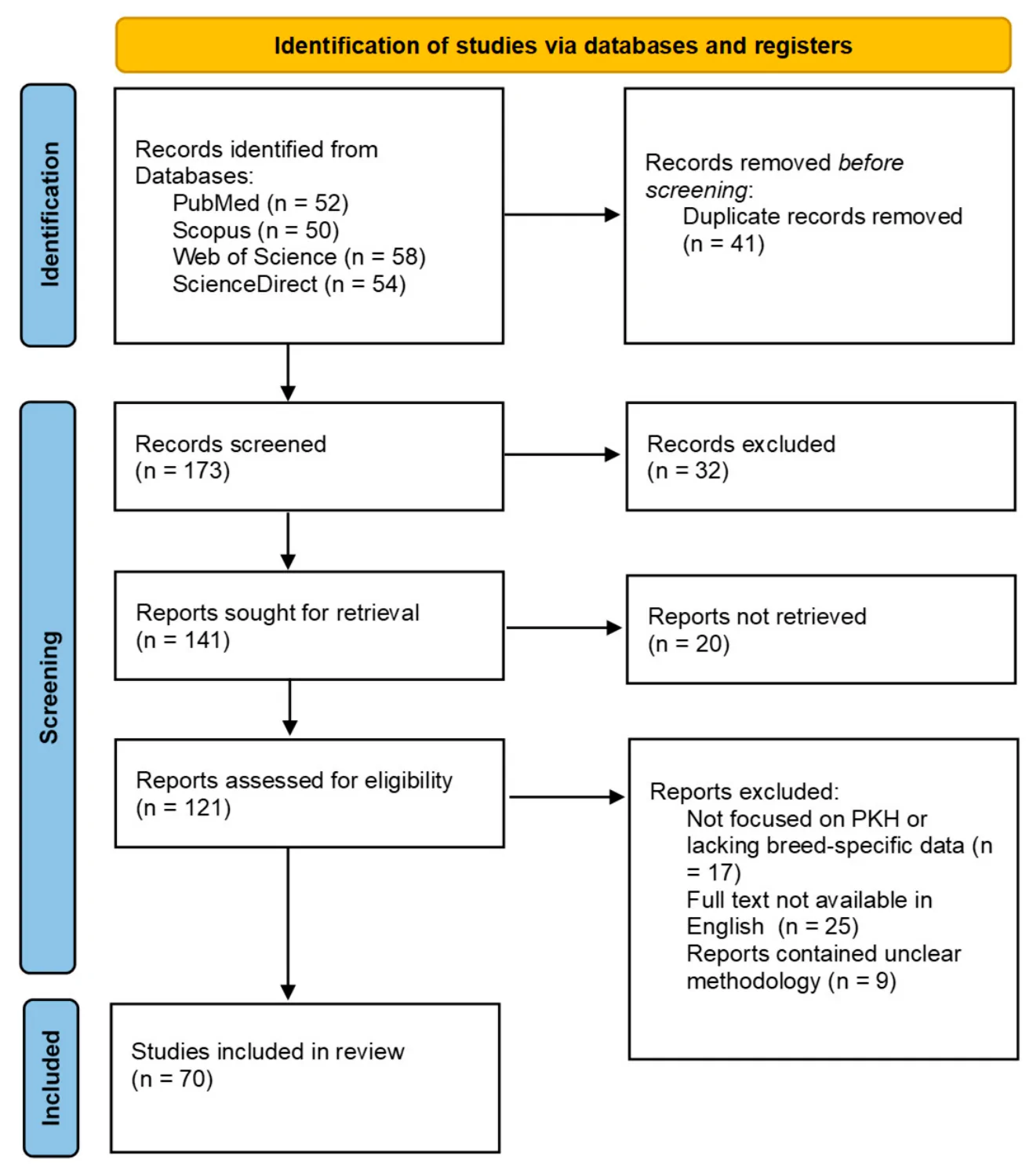
Records identified through database searching.

The search strategy (Figure 1) combined breed-specific terms with thematic keywords related to the principal research areas addressed in this review. The primary search string was based on the following breed-specific terms: (“Polish Konik” OR “Konik Polski” OR “Polish primitive horse” OR “Konik horse”). These terms were subsequently combined with thematic keywords using Boolean operators (AND), including:

- genetics,
- mitochondrial DNA,
- Y chromosome,
- behaviour,
- welfare,
- grazing,
- conservation grazing,
- rewilding,
- microbiome,
- reproduction,
- health,
- disease,
- usability,
- performance testing.

### 2.2. Eligibility Criteria

Studies were included if they met the following criteria:

Inclusion criteria:

- peer-reviewed scientific articles,
- original research papers and key review articles,
- publications released between 1 January 2005 and 31 May 2026,
- studies focused directly on PKH,
- studies including PKHs as comparative populations,
- publications written in English,
- studies containing clear methodological descriptions.

The review deliberately focused on English-language publications to ensure methodological consistency and international accessibility. Although numerous valuable studies concerning the PKH have been published in Polish, particularly in the fields of breeding history, reserve management and field performance testing, these publications were not systematically included because they are not indexed in the major international databases and are generally unavailable to the international scientific community. Selected historical publications were cited only when essential for understanding the development of the breed.

### 2.3. Narrative Synthesis

The literature selection process consisted of four stages:

- identification of records through database searching;
- removal of duplicate records;
- screening of titles and abstracts for relevance;
- full-text assessment according to the predefined eligibility criteria.

Because of substantial heterogeneity among study designs, investigated traits and analytical methods, a quantitative meta-analysis was considered inappropriate. Instead, the eligible publications were synthesised using a narrative approach and organised into thematic research areas.

The final review included 70 publications, which were classified into five principal themes:

- Origin and contemporary interpretation of the breed;
- Conservation genetics and founder line structure;
- Ecological function and conservation grazing;
- Health, reproduction and environmental adaptation;
- Behaviour, welfare and utility traits.

### 2.4. Declaration of Generative AI and AI-Assisted Technologies in the Manuscript Preparation Process

During the preparation of this work, the author used LeapSpace Elsevier v. 1.0, to conduct a literature search on ongoing genetic research and the use of the Polish Konik in landscape conservation. After using this tool/service, the author reviewed and edited the content as needed and takes full responsibility for the published article.

## 3. Origin and Contemporary Interpretation of the Polish Konik Horse

### From the Tarpan Hypothesis to Modern Genomics

The historical interpretation of the PKH has long been associated with the Eurasian tarpan (*Equus ferus ferus*) as the ancestral wild horse of Central and Eastern Europe. For decades, both scientific and popular literature described the PKH as either a direct descendant or a phenotypic reconstruction of the tarpan, largely on the basis of primitive morphology, dun coat colouration, dorsal stripe and adaptation to harsh environmental conditions [5,6,7]. This concept originated from the pioneering work of Tadeusz Vetulani, who initiated breeding programmes using primitive horses from the Biłgoraj region of southeastern Poland, believing that they retained phenotypic characteristics of the extinct tarpan [5,6,7]. Consequently, the early conservation programme focused predominantly on preserving primitive external features, environmental hardiness and maintaining semi-feral populations rather than improving utility or sport performance [41,42].

For several decades, this interpretation dominated both breeding philosophy and scientific research. As a consequence, the PKH was perceived primarily as a biological relic and a symbol of European natural heritage, while relatively little attention was devoted to its genetics, behaviour, health or practical utility [5,6]. However, rapid advances in molecular genetics and archaeogenomics over the last fifteen years have fundamentally changed our understanding of the breed’s origin.

Population genomic studies have demonstrated that the history of horse domestication was considerably more complex than previously assumed. Warmuth et al. [8] reconstructed patterns of horse domestication across Eurasia and showed extensive historical admixture among horse populations, providing little support for the persistence of isolated wild lineages in modern domestic breeds. Similarly, genome-wide SNP analyses conducted by Petersen et al. [9] demonstrated substantial genetic overlap between primitive and modern horse breeds, indicating that contemporary native breeds are part of the broader domestic horse gene pool rather than remnants of distinct wild populations.

The most significant breakthrough came from the archaeogenomic study of Librado et al. [12], who analysed 264 ancient horse genomes spanning the last 50,000 years. Their results demonstrated that virtually all modern domestic horses descend from a single ancestral population (DOM2) that originated in the western Eurasian steppes during the late fourth to early third millennium BCE and rapidly replaced most pre-existing horse populations throughout Eurasia. Importantly, these findings provided no evidence that the Polish Konik—or any other contemporary primitive breed—represents a surviving lineage of the Eurasian tarpan. Instead, the so-called tarpan itself appears to have originated through admixture between local European horses and horses belonging to the expanding DOM2 lineage, challenging the traditional view of the tarpan as the direct ancestor of modern domestic horses.

Recent whole-genome studies focusing specifically on the PKH further support this reinterpretation. Musiał et al. [14] demonstrated that the breed shares genomic components with ancient horses from the territory of present-day Poland and with other primitive European breeds, while also exhibiting evidence of historical admixture with modern domestic populations. Whole-genome sequencing, principal component analysis and admixture modelling consistently showed no closer relationship between the PKH and the published tarpan genome than that observed for other domestic breeds. The closest genomic affinity was identified with the Dülmen horse, which is readily explained by the documented use of Konik stallions in the post-war reconstruction of the Dülmen population rather than by shared wild ancestry [14].

These genomic findings are further supported by studies based on mitochondrial DNA and Y-chromosome markers. Cieślak et al. [33] demonstrated substantial mitochondrial haplotype diversity within the contemporary population, indicating that valuable maternal genetic variation has been preserved despite historical demographic bottlenecks. Similarly, Fornal et al. [10,11] and Musiał et al. [13] confirmed that both maternal and paternal founder structures remain identifiable within the present conservation population, although they differ in their interpretation of how founder lines should be defined and managed. While Cieślak and co-workers [33] proposed that maternal line classification should increasingly reflect mitochondrial haplotypes, Fornal and collaborators [10,11] argued that pedigree-based conservation remains fully justified because microsatellite analyses indicate considerable genetic coherence among the recognised genealogical lines. This discussion illustrates one of the most interesting contemporary challenges in conservation genetics, namely how traditional pedigree information should be integrated with rapidly expanding molecular evidence.

An important conceptual interpretation of these molecular findings was subsequently provided by Lovász et al. [43], who critically re-examined both historical sources and contemporary genetic evidence concerning the relationship between the PKH and the tarpan. The authors concluded that neither the existence of the tarpan as a clearly distinct ancestral species nor the direct descent of the PKH from wild European horses can currently be supported by available scientific evidence. Instead, they suggested that the enduring “Konik–tarpan” narrative reflects a combination of historical interpretation, conservation philosophy and cultural identity rather than genomic reality [43].

Taken together, the available evidence demonstrates a profound shift in the scientific perception of the PKH. Rather than representing a surviving relic of the Eurasian tarpan, the breed is now regarded as a native conservation population of complex domestic origin that combines a primitive phenotype with substantial genetic diversity, environmental adaptation and high conservation value [14]. Consequently, contemporary research has gradually moved beyond attempts to verify the tarpan hypothesis and now focuses on understanding the breed’s genetic diversity, behavioural adaptations, health, welfare, and ecological functions. This transition from a myth-based to an evidence-based interpretation represents one of the most important developments in PKH research over the last two decades.

## 4. Genetic Structure of Maternal and Paternal Lines

The PKH represents a relatively small conservation population that was reconstructed following a severe demographic decline during the twentieth century. Consequently, monitoring genetic diversity and founder-line integrity has become one of the principal objectives of conservation breeding programmes [11,44]. Genetic diversity in conservation populations is currently evaluated using complementary molecular approaches that provide information on different components of population structure. Mitochondrial DNA (mtDNA), inherited exclusively through the maternal line, is widely used to identify maternal haplotypes and assess the continuity of founder mare families. In contrast, Y-chromosome markers characterise paternal inheritance and are particularly useful for monitoring the diversity of stallion lines. Nuclear microsatellite markers and, more recently, genome-wide single-nucleotide polymorphism (SNP) analyses provide information on overall genetic variability, population structure, inbreeding, and relationships among individuals and populations. Parameters such as observed and expected heterozygosity, allelic richness, and fixation indices (FST) are commonly used to evaluate the genetic status of conservation populations and to support breeding decisions aimed at maintaining long-term genetic diversity.

Studies on the PKH have employed these complementary approaches to address different aspects of the breed’s genetic structure. Consequently, interpretation of the available evidence requires consideration of the biological information provided by each marker system, as different molecular methods describe distinct components of population history and genetic diversity rather than representing competing measures of conservation value.

During the last two decades, molecular techniques, including mitochondrial DNA sequencing, microsatellite markers, Y-chromosome analyses and, more recently, whole-genome sequencing, have considerably improved understanding of the breed’s genetic structure [10,11,13,14,33]. The first studbook, published in 1962, included 35 maternal and 6 paternal founder lines; however, 19 maternal lines subsequently became extinct [11]. Although the contemporary population derives from a limited number of founders and experienced a substantial demographic bottleneck during the twentieth century, available studies consistently indicate relatively favourable levels of heterozygosity and genetic diversity, as well as limited genomic inbreeding [10,11,44].

The methods used in these studies provide information at different levels. Pedigree records document the continuity and relative contribution of recognised founder families. Mitochondrial DNA describes maternal ancestry, whereas Y-chromosome markers identify paternal relationships. Microsatellites characterise nuclear genetic diversity, population differentiation and inbreeding, while whole-genome sequencing provides a higher-resolution assessment of population structure and broader evolutionary relationships [10,11,13,14,33]. The main contributions and limitations of these approaches are summarised in Table 2.

### 4.1. Maternal Lines

The presence of maternal diversity is particularly important in small conservation populations because mtDNA diversity may be lost rapidly following demographic decline or unequal contributions to breeding among mare families.

Maternal diversity in the PKH has been investigated primarily by analysing the mitochondrial D-loop, which is inherited through the maternal lineage and does not undergo recombination. Consequently, mtDNA is particularly useful for tracing founder mares, identifying maternal haplotypes and testing the consistency of pedigree-defined female lines [33]. However, the correspondence between mtDNA haplotypes and pedigree-defined maternal lines was incomplete. Several genealogical lines contained more than one mitochondrial haplotype, whereas identical haplotypes occurred in horses assigned to different founder lines. These discrepancies suggested inaccuracies in historical pedigrees or previously unrecognised relationships and led the authors to propose reconsidering maternal-line classification using molecular evidence [33].

A different but complementary picture was obtained from nuclear microsatellite markers. Fornal et al. [12] analysed representatives of the surviving maternal lines and reported high allelic richness and expected heterozygosity, accompanied by low inbreeding. Bayesian population-structure analysis distinguished three partially differentiated genetic clusters, but these clusters did not correspond strictly to the recognised genealogical lines. The findings indicated extensive historical gene flow among mare families and suggested that the currently recognised lines should not be interpreted as genetically isolated subpopulations [12].

Musiał et al. [13] subsequently combined mtDNA and Y-chromosome data, confirming substantial maternal haplotype diversity and showing that the breed’s molecular structure cannot be reduced to genealogical line assignment alone. The study identified 43 maternal haplotypes and demonstrated that several haplotypes were shared across pedigree-defined lines, whereas some lines contained multiple haplotypes [13]. More recent collaborative research has further supported the view that genealogical and molecular information describe different, but complementary, components of maternal population structure rather than mutually exclusive classifications [34].

Collectively, these studies demonstrate that the PKH has retained considerable maternal diversity, but they also show that a genealogical line does not necessarily represent a genetically uniform maternal unit. Pedigree records remain essential for preserving historical breeding continuity, whereas mtDNA can reveal hidden relationships, pedigree inconsistencies and rare maternal variants. The practical conservation value of these approaches therefore lies in their combined use rather than in replacing one classification system with another.

### 4.2. Stallion Lines

Paternal diversity is considerably narrower than maternal diversity because the contemporary PKH population derives from six recognised founder sire lines. This pattern reflects both the restricted number of male founders and the unequal reproductive contribution typical of stallions in managed horse populations.

Using microsatellite markers, Fornal et al. [10] examined individuals representing all six male founder lines. The study demonstrated high overall genetic variability but only weak differentiation among sire lines. Low F_ST values and substantial admixture among the analysed groups indicated that the genealogical sire lines do not constitute separate nuclear genetic subpopulations [10]. Nevertheless, differences in the frequency and reproductive contribution of particular lines remain relevant because disproportionate use of individual stallions may accelerate the loss of rare paternal variation.

Y-chromosome analysis provided a more direct assessment of paternal ancestry. Musiał et al. [13] detected limited Y-chromosomal diversity and identified haplotypes shared not only among different PKH sire lines but also with other European breeds. The Y-chromosome results therefore did not reproduce the six genealogical lines as six genetically distinct paternal groups. Instead, they suggested a narrower and partly shared paternal background, consistent with the limited diversity commonly observed in domestic horse Y chromosomes [13].

Whole-genome analysis confirmed that PKHs form a compact genetic cluster, but it did not identify genomic divisions corresponding to the recognised sire lines [14]. Accordingly, the six sire lines retain substantial historical and breeding importance, although their classification should not be interpreted as evidence of six distinct genomic lineages. Their conservation remains justified primarily as a means of balancing founder contributions and reducing the risk that individual paternal branches will disappear from the breeding population.

### 4.3. Conservation Genetics Implications

Pedigree and molecular studies provide a generally favourable assessment of genetic variation in the contemporary PKH population. Microsatellite analyses revealed relatively high heterozygosity and allelic richness, while estimates based on pedigree and genomic data indicated that inbreeding has remained limited despite the restricted founder base [10,11,44]. These findings suggest that coordinated mating and the maintenance of multiple breeding centres have reduced the rate of genetic erosion.

Nevertheless, favourable population-level diversity does not eliminate vulnerability at the lineage level. Nineteen of the original 35 maternal lines have disappeared; the paternal population is derived from only six recognised sire lines; and founder contributions are unequal [10,11]. Consequently, an increase in total population size does not necessarily guarantee preservation of rare genealogical or molecular variants. Particular attention should therefore be paid to under-represented families, unequal stallion use and the potential loss of uncommon mtDNA or Y-chromosome haplotypes.

The available evidence also indicates that no single method fully captures the breed’s conservation status. Pedigree analyses are indispensable for documenting founder representation and controlling planned matings, but their reliability depends on the completeness and accuracy of historical records. Mitochondrial and Y-chromosome markers provide direct information on uniparental ancestry, although they represent only a small proportion of the genome. Microsatellites assess nuclear diversity and relatedness but offer lower resolution than genome-wide markers. Whole-genome sequencing provides the most comprehensive view, although current conclusions remain limited by relatively small and unevenly represented samples [10,11,13,14].

Thus, the principal contribution of recent genetic research is not the replacement of genealogical lines with a new molecular classification, but a more precise understanding of what each classification represents. Further research is needed to determine how pedigree information and molecular data may complement each other in future conservation monitoring of the PKH. Such a cautious, integrated approach may support the preservation of overall diversity, rare founder contributions and the historical continuity of the breed without treating genealogical lines as genetically isolated populations.

## 5. Reproductive Biology and Adaptation

Reproductive efficiency is widely recognised as a characteristic biological feature of the PKH. Unlike highly selected sport horse breeds, where reproduction is frequently supported by intensive reproductive management, Polish Koniks have retained high fertility under predominantly natural mating systems maintained in both stable-based and semi-feral conditions. Long-term natural selection, together with conservation-oriented breeding, has favoured fertility, maternal ability, reproductive longevity and adaptation to fluctuating environmental conditions, making reproductive success one of the defining characteristics of the breed [29,30].

The high reproductive performance of the PKH has been consistently documented under practical breeding conditions. In a 17-year study conducted in the Popielno conservation herd, conception and pregnancy rates exceeded 90%, whereas foaling rates approached 86%. More than two-thirds of mares conceived during the first oestrous cycle, while embryonic loss and twin pregnancies occurred in 0,96% and 1,29%, respectively [29]. The consistently high reproductive performance of the PKH appears to depend not only on physiological fertility but also on behavioural characteristics that facilitate successful natural reproduction. In a comparative study of Polish Konik and Thoroughbred mares, Górecka et al. [20] demonstrated that Konik mares exhibited significantly more pronounced behavioural signs of oestrus and achieved markedly higher pregnancy rates (88.5% vs. 46.0%). The authors suggested that the clearer expression of oestrous behaviour and the lower emotional reactivity characteristic of this primitive breed may improve reproductive communication between mares and stallions, thereby increasing mating success under natural breeding conditions [20]. These findings indicate that behavioural stability may represent an important, although often overlooked, component of reproductive fitness in the PKH and illustrate the close relationship between temperament, reproductive behaviour and long-term adaptation to extensive management systems.

Studies performed in semi-feral populations have further demonstrated that reproductive success in the PKH depends not only on fertility itself but also on stable social organisation. Long-term observations of free-ranging harems showed that replacement of dominant stallions did not adversely affect pregnancy rates, foal survival or mare reproductive performance [30]. Neither infanticide nor increased pregnancy loss associated with harem stallion succession was observed, providing evidence against the occurrence of Bruce-effect-like mechanisms in this species [30]. These findings indicate that the social structure of semi-feral Konik populations provides a remarkably stable reproductive environment, supporting long-term population persistence.

Environmental conditions also play a significant role in regulating reproductive physiology. Opałka et al. [45] demonstrated pronounced seasonal fluctuations in androgen concentrations in Polish Konik stallions, with endocrine profiles differing between animals kept in stables and those free-ranging. Stallions maintained under reserve conditions exhibited hormonal patterns that more closely reflected natural reproductive seasonality, suggesting that management practices may influence reproductive endocrinology even within the same breed.

An additional area of growing interest concerns the interaction between reproduction, immunity and environmental adaptation. Studies conducted on pregnant Polish Konik mares demonstrated that the management system influences cell-mediated immune responses during the periparturient period. Compared with mares maintained under conventional stable conditions, mares living in a reserve exhibited a consistently higher CD4:CD8 lymphocyte ratio throughout the periparturient period, accompanied by a transient reduction in CD8+ lymphocytes immediately after foaling. These observations indicate that environmental conditions may modulate cellular immune responses during the periparturient period, supporting the concept that physiological adaptation in the PKH extends beyond reproduction to include immunological regulation [39]. Complementary studies on the major histocompatibility complex (MHC) revealed substantial immunogenetic diversity within the breed, suggesting that maintenance of MHC variability may contribute not only to disease resistance but also to reproductive fitness and long-term population viability [31].

Collectively, the evidence confirmed a linkage that the exceptional reproductive performance of the PKH reflects a complex interaction among behavioural stability, seasonal reproductive physiology, maternal adaptation, immunological competence, and genetic diversity, rather than fertility alone. Consequently, reproduction in PKH appears to be an adaptive biological trait shaped by long-term natural selection and selection aimed at species conservation. The positive influence and importance of long-term natural and conservation-oriented selection on adaptive biological traits should be considered and explored. This integrative perspective distinguishes the breed from many intensively managed horse populations and further supports its value as a model for studies on reproductive biology, environmental adaptation and conservation breeding.

## 6. Ecological Function and Conservation Grazing

In recent decades, the ecological role of the PKHs has become one of the most intensively investigated aspects of the breed. Initially maintained primarily as a primitive-type conservation population, PKHs are now widely used in semi-feral grazing systems to maintain open habitats, limit shrub encroachment, and support biodiversity in semi-natural ecosystems [15,17,18]. Owing to their environmental hardiness, ability to use low-quality forage and adaptation to year-round outdoor management, PKHs have become one of the principal large herbivores used in conservation grazing programmes in Central Europe [4,6]. Compared with intensive livestock systems, conservation grazing involving PKHs is typically characterised by low-input management and long-term grazing in wetlands, river valleys, forest clearings, and abandoned agricultural landscapes [15]. Such systems allow horses to express natural behavioural patterns while simultaneously influencing vegetation structure, nutrient redistribution and habitat heterogeneity.

### 6.1. Semi-Feral Systems

Semi-feral management systems constitute one of the defining characteristics of the PKH population. In Poland, such systems are maintained particularly in nature reserves, national parks and ecological restoration projects, including Popielno, Roztocze, the Biebrza Valley and several wetland conservation areas [15,17,18,26]. Unlike stable-based horse management, semi-feral systems expose animals to fluctuating environmental conditions, seasonal variation in forage availability and unrestricted social interactions. These systems therefore provide a unique opportunity to investigate long-term adaptation of domestic horses to extensive environmental conditions [26]. At the same time, semi-feral management allows maintenance of natural social structures, locomotor activity and foraging behaviour, which are often reduced in conventional horse husbandry systems [26].

Studies conducted on PKH populations demonstrated that the breed exhibits substantial adaptation to year-round outdoor management, including efficient utilisation of poor-quality forage, seasonal metabolic adjustment and relatively stable social organisation [15,26,32]. These features likely reflect both historical selection under extensive conditions and long-term conservation breeding focused on preserving primitive adaptive traits. Nevertheless, semi-feral management also creates welfare and management challenges associated with winter food limitation, parasite burden, injuries and monitoring of population size [26]. Studies in nature reserves confirm high diversity of intestinal parasites, including rare nematode species [46,47] and tapeworms [48]. Consequently, conservation grazing systems involving PKHs require continuous balancing between ecological objectives and animal welfare considerations.

### 6.2. Vegetation and Landscape Shaping

One of the most important ecological functions of PKHs involves their influence on vegetation structure and landscape dynamics. Grazing by large herbivores contributes to habitat heterogeneity through selective feeding, trampling, and nutrient redistribution, thereby preventing excessive vegetation succession and maintaining open landscapes [4,49]. Studies conducted in conservation grazing systems demonstrated that PKHs effectively reduce shrub encroachment and limit the expansion of woody vegetation in abandoned or semi-natural habitats [15,18]. Grazing also contributes to the creation of a mosaic vegetation structure characterised by patches of short grassland, taller vegetation, and disturbed microhabitats. Such heterogeneity may increase habitat availability for numerous plant and animal species. Importantly, the ecological effects of PKH grazing depend strongly on stocking density, habitat type, and seasonal environmental conditions. Moderate grazing intensity appears particularly beneficial because it maintains disturbance without causing severe overgrazing or soil degradation [18]. In contrast, excessively high grazing pressure may negatively affect vegetation composition and habitat quality.

GPS-based ecological studies additionally demonstrated that PKHs preferentially utilise open habitats and wetland-associated vegetation while avoiding densely forested areas [16,17]. These habitat preferences significantly influence spatial grazing patterns and contribute to uneven but ecologically valuable landscape disturbance. Conservation grazing by large herbivores is currently regarded as an important tool for maintaining open habitats and preventing secondary succession in many European ecosystems [3,4]. In wetlands and river valleys, grazing helps preserve open sedge meadows, marsh vegetation, and ecotonal habitats that support numerous bird, invertebrate, and plant species. One of the most important practical applications of PKHs in conservation management concerns wetland protection projects conducted in northeastern Poland, particularly in the Narew and Biebrza river valleys. Following the abandonment of traditional mowing and grazing systems in the second half of the twentieth century, many marsh habitats experienced rapid shrub and tree encroachment, resulting in the progressive loss of open fen and wet meadow ecosystems important for breeding wader and waterbird species [15,17,50]. Field observations from marsh habitats indicated that grazing helped maintain open vegetation structure required by numerous wetland bird species, including northern lapwing (*Vanellus vanellus*), common snipe (*Gallinago gallinago*), great snipe (*Gallinago media*), black-tailed godwit (*Limosa limosa*) and Eurasian curlew (*Numenius arquata*) [15]. In unmanaged areas, progressive shrub succession led to habitat transformation and a decline in characteristic open-marsh avifauna [15].

Importantly, studies demonstrated that the ecological effects of grazing were strongly dependent on grazing intensity, season and hydrological conditions. Autumn and winter grazing appeared particularly effective at reducing shrub expansion in marsh ecosystems [50,51].

### 6.3. Rewilding Context

The Oostvaardersplassen reserve has become one of the best-known European examples of trophic rewilding, in which PKH, Heck cattle and red deer were introduced to restore natural grazing processes and maintain open wetland and grassland habitats [3]. Ecological studies have demonstrated that large herbivores contribute to vegetation dynamics, habitat heterogeneity, and the maintenance of open landscapes, making the reserve an important reference site for conservation grazing research. Despite these ecological achievements, the management strategy adopted at Oostvaardersplassen generated considerable ethical debate. Repeated episodes of winter food limitation and high mortality among large herbivores, particularly during 2005 and 2017–2018, raised concerns regarding animal welfare in fenced rewilding systems lacking natural predators and opportunities for seasonal migration [52,53,54]. These discussions highlighted the importance of balancing ecological objectives with adaptive population management and welfare monitoring, rather than questioning the ecological role of large herbivores per se. Consequently, contemporary conservation grazing programmes increasingly adopt adaptive management strategies that integrate ecological objectives with regular monitoring of population size, habitat carrying capacity and seasonal nutritional conditions. Within this framework, the PKH is best regarded not as a reconstructed wild horse, but as a native conservation breed whose ecological significance lies in its ability to restore and maintain dynamic semi-natural ecosystems while remaining subject to evidence-based welfare management.

## 7. Environmental Sensitivity and Management-Related Disorders

Although the PKH is widely regarded as a hardy and environmentally resilient breed, increasing evidence suggests that its evolutionary adaptation to extensive management systems may simultaneously predispose it to specific disorders when maintained under modern husbandry conditions. Unlike highly managed sport horse populations, Polish Koniks evolved under conditions characterised by year-round outdoor housing, natural ventilation, seasonal fluctuations in forage availability and prolonged locomotor activity associated with extensive grazing. Consequently, an abrupt transition to intensive, stable management, reduced environmental variability, and continuous access to energy-rich diets may create a biological mismatch between the breed’s evolutionary adaptations and contemporary management practices. Among the disorders most closely associated with this phenomenon are equine asthma and equine metabolic syndrome (EMS).

### 7.1. Equine Asthma as a Consequence of Environmental Change

Equine asthma is one of the best-documented examples of environmental sensitivity in the PKH. The disease is now recognised as a multifactorial inflammatory disorder of the lower respiratory tract in which chronic exposure to organic dust, fungal spores, mites and endotoxins constitutes the principal initiating factor [55,56,57,58]. Stable-related factors, particularly poor ventilation and feeding mould-contaminated or poorly preserved hay, substantially increase the risk of disease development [55,56,57,58].

One of the first studies focusing specifically on the PKH was conducted by Niedźwiedź and Jaworski [22], who described recurrent respiratory disease in horses maintained under both pasture and stable conditions. Their observations demonstrated that despite the breed’s reputation for environmental hardiness, chronic inflammatory airway disease may develop in horses exposed to unfavourable stable environments. Subsequent epidemiological studies by Borowska et al. [23] further demonstrated that maternal inbreeding coefficients were associated with an increased risk of asthma, whereas individual inbreeding coefficients were not. Importantly, the authors emphasised that management-related factors, including air quality, stable ventilation and exposure to organic dust, remained the dominant determinants of disease occurrence [23].

Additional support for the importance of environmental exposure comes from studies investigating airborne particulate matter in horse stables. The respirable dust particles, particularly those with aerodynamic diameters below 5 μm, readily penetrate the lower airways and may contribute to the initiation and persistence of chronic airway inflammation [59]. These observations complement previous clinical studies by highlighting the importance of stable microclimate and airborne particulate exposure in the pathogenesis of equine asthma.

Collectively, the available evidence indicates that asthma in the PKH is primarily a consequence of environmental conditions that differ substantially from those under which the breed evolved. Respiratory disease appears to result from prolonged exposure to enclosed housing, inadequate ventilation and inhalation of organic dust generated by conserved forage. This interpretation is fully consistent with the contemporary understanding of equine asthma as a management-associated disease and supports the use of preventive strategies based primarily on environmental modification rather than pharmacological intervention alone [60].

### 7.2. Metabolic Adaptation and Equine Metabolic Syndrome

A similar concept of environmental mismatch may explain the increasing recognition of metabolic disorders in primitive horse breeds. Throughout their evolutionary history, Polish Koniks were exposed to marked seasonal fluctuations in forage availability, prolonged periods of nutritional limitation, and extensive daily locomotor activity associated with free-ranging grazing systems [61,62]. Under these conditions, efficient energy utilisation represented a clear adaptive advantage [32].

Modern management systems differ substantially from these historical conditions. Continuous access to energy-rich forage, highly productive pastures and concentrate feeding, combined with reduced physical activity, may predispose primitive breeds to obesity, insulin dysregulation and equine metabolic syndrome (EMS) [40,61]. EMS is characterised by insulin dysregulation, hyperinsulinaemia and an increased risk of endocrinopathic laminitis, which has become one of the most important welfare concerns in native and pony-type breeds [40,62].

### 7.3. Environmental Adaptation and Future Health Management

Several reports suggest that primitive and metabolically efficient horse breeds may exhibit increased susceptibility to insulin dysregulation under intensive feeding conditions, similar to pony breeds and other “easy keeper” populations [61,62]. This interpretation is consistent with the concept of a “thrifty genotype”, in which animals evolutionarily adapted to fluctuating nutritional availability may develop metabolic dysfunction when exposed to chronic caloric excess and reduced environmental variability. A similar metabolic predisposition associated with modern feeding systems has also been observed in other primitive and native horse populations, including Icelandic horses and pony breeds, in which obesity and insulin dysregulation are increasingly recognised as important welfare and management challenges [61,62]. Consequently, the maintenance of appropriate body condition, controlled nutrition, and sufficient locomotor activity should constitute important components of welfare-oriented management in PKHs, particularly in non-reserve populations maintained in stabling.

It can therefore be assumed that the underlying cause of the two conditions mentioned, particularly metabolic syndrome, lies not in any specific breed predisposition among horses, but in the mismatch in environmental conditions. Both appear to arise when animals that evolved in extensive grazing systems are maintained under conditions characterised by restricted movement, limited environmental variability and excessive nutritional supply. Consequently, these disorders should be interpreted not only as clinical diseases but also as indicators of a mismatch between the breed’s evolutionary adaptations and contemporary husbandry practices.

This perspective reinforces one of the central conclusions emerging from the present review. The biological characteristics that make the PKH an exceptionally valuable breed for conservation grazing—including its hardiness, metabolic efficiency and adaptation to extensive environments—may simultaneously increase its susceptibility to management-related disorders when traditional environmental conditions are replaced by intensive stable husbandry. Therefore, preserving the health and welfare of the breed requires management strategies that take into account not only veterinary recommendations but also the ecological and evolutionary context in which the PKH developed.

## 8. Behaviour, Welfare and Utility

Behavioural time budgets have been proposed as robust animal-based indicators of equine welfare, since horses maintained under conditions that allow natural foraging, locomotion, and social interactions exhibit activity patterns that more closely resemble those of free-ranging populations [63]. Behavioural characteristics, emotional reactivity, and adaptation to semi-feral management are among the most extensively investigated aspects of the PKH [21,24]. Management systems, early environmental exposure, and semi-feral upbringings substantially influence emotional reactivity, stress responses, and social behaviour in the breed [24]. Available studies suggest that the breed combines relatively low emotional reactivity with good adaptation to extensive systems and stable social behaviour [30], although semi-feral management may also generate specific welfare challenges associated with environmental exposure and management interventions [26]. Welfare protocols developed for horses kept in traditional systems need to be adapted for horses living in reserve conditions [28].

### 8.1. Temperament, Character and Emotional Reactivity

Temperament, character and emotional reactivity are key behavioural traits that influence horse welfare, trainability, human–animal interactions, and utility value. In PKHs, particular attention has been directed toward behavioural stability and adaptation to handling following semi-feral upbringing. Górecka-Bruzda and collaborators assessed the aversiveness and effects on the horse-human relationship of several routine procedures in pre-weaned PKH foals [25]. They revealed that even after eliciting avoidance behaviour and inducing activation of certain physiological stress indicators (i.e., an increase in cortisol levels), these effects were transient and did not alter human perception following the procedures [25]. Studies on PKHs suggest that extensive management and early environmental experience are associated with stable behavioural responses and relatively low emotional reactivity [25]. In contrast, temperament and emotional reactivity are recognised as important determinants of training and performance in sport horses, where behavioural responses are influenced by both genetic background and management practices [64,65]. Behavioural traits may have a particularly strong influence on both rider safety and the overall assessment of the horse. Therefore, temperament and character should be regarded as key breeding objectives alongside the preservation of primitive breed characteristics. Kozak & Budzynska [36] reported high scores during routine handling procedures, with many horses receiving maximum scores during brushing and other human–horse interactions. Such findings support the view that PKHs generally display favourable behavioural characteristics and a high willingness to cooperate with humans [36]. Behavioural traits associated with utility performance testing were investigated in PKHs. Their analyses demonstrated that several behavioural components currently used in performance evaluation systems show relatively limited variability, suggesting substantial behavioural uniformity within the breed [35]. The authors additionally emphasised that traits such as behaviour, effort tolerance and recovery parameters may require modified scoring systems to improve their usefulness in breeding selection.

The favourable behavioural profile of the PKH has also contributed to its increasing use in equine-assisted interventions. Comparative studies have demonstrated that both the Polish Konik and the Hucul horse possess behavioural characteristics desirable for hippotherapy, including a calm temperament, predictable responses to external stimuli, and good cooperation with humans [37].

Behavioural stability may also contribute to the breed’s exceptional reproductive performance. In a comparative study of Polish Konik and Thoroughbred mares, Konik mares exhibited significantly more pronounced behavioural signs of oestrus and achieved substantially higher pregnancy rates [20]. The authors suggested that the clearer expression of oestrous behaviour and lower emotional reactivity characteristic of this primitive breed may facilitate reproductive communication between mares and stallions, thereby improving mating success under natural breeding conditions [20]. These findings illustrate the close relationship between temperament, reproductive biology and adaptation to extensive management systems.

### 8.2. Social Behaviour in Semi-Feral Systems

**Figure 2:**
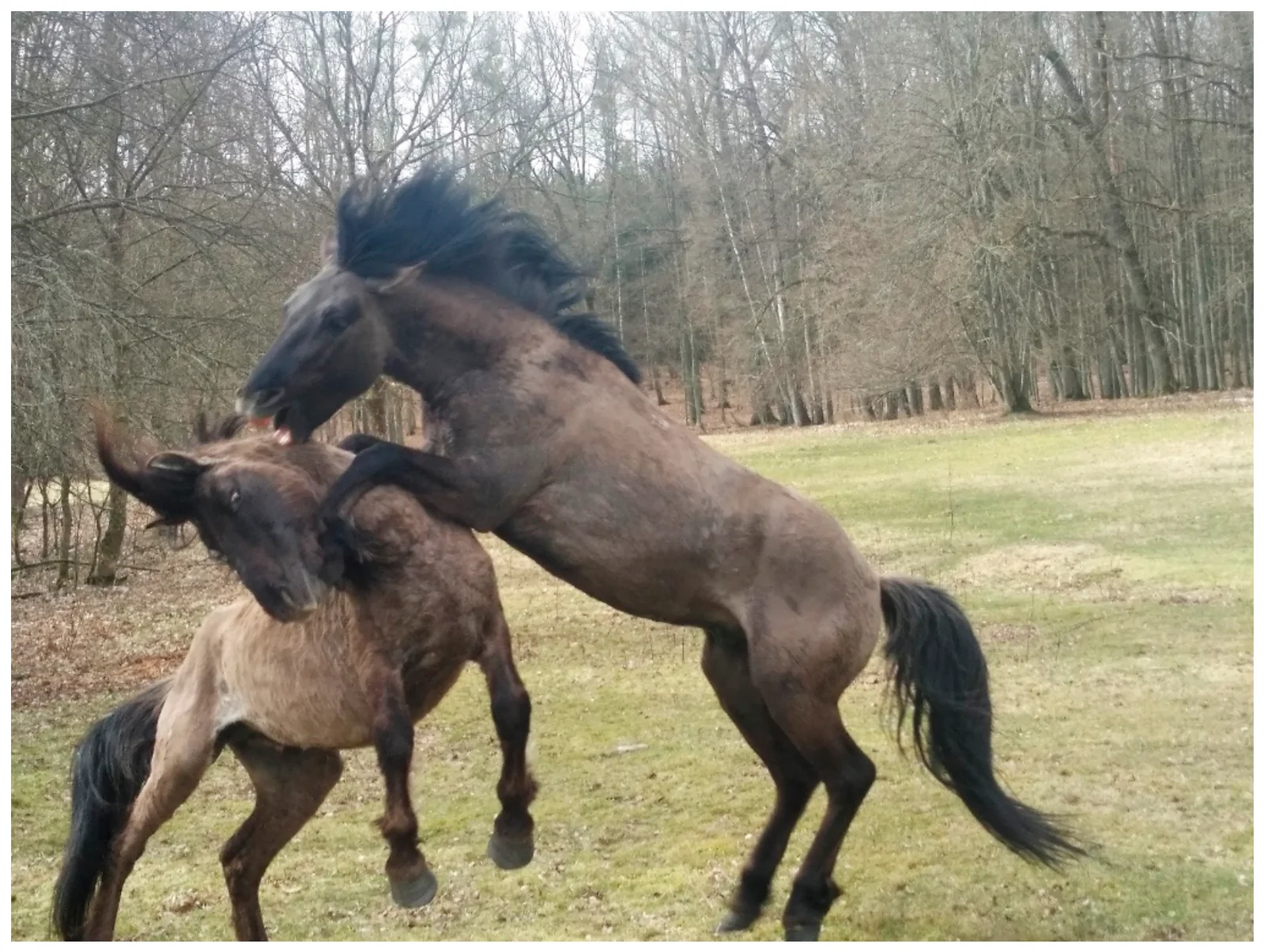
A fight between the stallions Mor (22 years old) and Turbacz (3 years old) to take the harem away from the older stallion at the Popielno reserve (photo by the author).

Semi-feral populations of PKHs provide a unique model for investigating social organisation and behavioural adaptation in domestic horses maintained under low-intervention environmental conditions. Unlike conventionally stabled horse populations, PKHs maintained in reserve and free-ranging systems can exhibit a broad spectrum of natural social behaviours, including harem formation and fights between stallions (Figure 2), long-term mare–foal interactions, social grooming, avoidance behaviour, and collective movement patterns [26,38]. Recent quantitative evidence has demonstrated that management conditions markedly influence the equine time-activity budget, with free-ranging horses exhibiting longer feeding times, increased locomotion, and behavioural patterns that more closely resemble their natural repertoire than those of horses maintained under intensive housing conditions [66].

Studies performed on semi-feral PKH populations demonstrated relatively stable social hierarchy and long-term group cohesion, particularly within mare–offspring groups and harem systems controlled by adult stallions [26,30]. Comparable patterns of social organisation have been described in other primitive and free-ranging horse populations, including Sorraia horses and the feral Cavalli di Esperia [67,68], suggesting that stable harem organisation represents a conserved feature of equine social systems. Within these groups, social relationships established during the first months of life are thought to play an important role in behavioural development and subsequent responses to environmental challenges. Consequently, several studies have investigated how early-life management influences behavioural adaptation in Polish Konik foals. Górecka-Bruzda et al. [24] demonstrated that foals raised under forest reserve conditions exhibited distinct physiological and behavioural responses during weaning compared with stable-reared animals. These findings indicate that early environmental experience may shape stress responsiveness and behavioural adaptation later in life. Importantly, studies on semi-feral harem systems indicated that the social organisation of PKHs remains relatively stable despite periodic changes in stallion structure or group composition [30]. Such observations support the hypothesis that the breed retains substantial behavioural adaptability associated with long-term maintenance under extensive conditions. Similar social structures involving harem organisation, female–offspring groups and stallion-mediated social stability have also been described in other free-ranging horse populations, including Sorraia horses and feral horse systems studied in Europe and Asia [68]. Conversely, the larger the group, the less stable its social structure becomes. In such cases, the dominant stallion has greater difficulty maintaining his position in the herd, and in large groups, a tendency to form subgroups is often observed [67].

## 9. Future Perspectives

Although considerable progress has been made in understanding the biology and conservation of the PKH, several important research priorities remain.

One of the most promising directions is the broader application of whole-genome sequencing (WGS) and high-density genomic analyses in conservation breeding. While pedigree records remain the foundation of the current breeding programme, genome-wide approaches could improve the identification of cryptic relatedness, quantify genomic inbreeding more accurately than pedigree-based estimates, and help preserve both neutral and adaptive genetic diversity. Future studies integrating pedigree information with genomic data may therefore optimise breeding decisions while maintaining the breed’s characteristic founder-line structure.

Another important challenge concerns the implementation of genomic-informed conservation management. Rather than focusing solely on minimising inbreeding coefficients, future breeding programmes could incorporate genomic estimates of effective population size, genomic relationships and adaptive genetic variation, allowing more precise management of small conservation populations.

Another promising area of research concerns the microbiota of anatomically distinct body sites and its role in health and adaptation. Recent studies in PKH have characterised the ocular surface microbiome in foals and the vaginal microbiome in postpartum mares [69,70]. These investigations provide an important foundation for future studies exploring the functional relationships between host-associated microbial communities, immune responses, reproductive health and environmental adaptation. Expanding this research through culture-independent approaches, including metagenomic and metabolomic analyses, may provide a more comprehensive understanding of host–microbiota interactions in native horse breeds.

Climate change is likely to become an increasingly important factor influencing the management of native horse breeds. Rising temperatures, altered precipitation patterns and changes in vegetation composition may affect forage availability, parasite dynamics and wetland ecosystems in which semi-feral Polish Konik populations are maintained. Long-term monitoring programmes combining ecological, physiological and genetic data will therefore be essential for evaluating the resilience of conservation populations under changing environmental conditions.

Future welfare assessment would also benefit from integrating precision livestock technologies. GPS tracking, accelerometry, remote sensing, automated behavioural monitoring and non-invasive physiological measurements could provide continuous information on movement patterns, habitat use, body condition and health in free-ranging horses. Such approaches would allow welfare assessment to be integrated with ecological monitoring while minimising disturbance to semi-feral populations.

Finally, one of the greatest opportunities for future research lies in integrating genetics, ecology, behaviour, health and welfare within multidisciplinary study designs. Such an approach would improve our understanding of how evolutionary adaptation, environmental conditions and conservation management interact to shape the biology of the PKH and would support evidence-based conservation strategies for this and other native horse breeds.

## 10. Conclusions

The PKH represents far more than a symbolic descendant of the Eurasian tarpan. Contemporary research has established the breed as an important model for studies on conservation genetics, environmental adaptation, behavioural biology and conservation grazing. Although historical interpretations emphasised its presumed wild ancestry, current evidence indicates that the breed’s scientific value lies primarily in its unique combination of genetic diversity, ecological functionality, and adaptation to extensive management systems.

The literature reviewed demonstrates that the PKH has successfully retained substantial genetic variability despite its restricted founder population, while modern molecular approaches have provided new insights into the relationship between pedigree structure and genetic diversity. At the same time, ecological studies have shown that the breed plays an important role in maintaining habitat heterogeneity and supporting biodiversity in semi-natural ecosystems.

Behavioural and physiological investigations further indicate that many of the breed’s characteristic traits—including low emotional reactivity, high reproductive efficiency and environmental resilience—are closely linked to its long-term adaptation to extensive management systems. Conversely, disorders such as equine asthma and equine metabolic syndrome illustrate how modern husbandry practices may create a mismatch between the breed’s evolutionary adaptations and contemporary management conditions.

Despite the considerable progress achieved over the past two decades, important knowledge gaps remain. In particular, further studies integrating whole-genome analyses, functional microbiome research, long-term welfare assessment and precision monitoring of semi-feral populations are needed to better understand the mechanisms underlying adaptation and to support evidence-based conservation breeding of this unique native horse breed.

## Figures and Tables

**Table 1:** Evolution of Scientific Perspectives on the PKH.

Research Area	Traditional View	Current Evidence	Key References
Origin	Direct descendant of the Tarpan	Complex origin; no genomic evidence for direct descent from the Tarpan	[5,6,7,12,14,32]
Genetic structure	Founder lines defined by pedigree records	Complex genetic structure revealed by mtDNA, microsatellites and WGS	[11,12,14,33,34]
Ecological role	Primitive horse maintained in reserves	Ecosystem engineer used in conservation grazing and habitat restoration	[15,17,18]
Behaviour	Primitive, hardy horse	Model for studies of emotional reactivity, welfare and social behaviour	[21,24,26,27,28,35,36,37,38]
Reproduction	Naturally fertile breed	Well-characterised reproductive physiology in a few aspects and adaptation to semi-feral systems	[29,30,31,32,39]
Health	Generally resistant breed	Environmentally sensitive to management-related disorders (asthma, EMS, laminitis)	[22,23,40]

**Table 2:** Molecular and genealogical approaches for investigating genetic diversity in the PKH.

Approach	Information Obtained	Principal Findings in the PKH	Main Limitations	Representative References
Pedigree analysis	Founder-line continuity, relationships and founder contributions	Documentation of recognised maternal and paternal lines; identification of unequal family contributions and lost lines	Dependent on completeness and accuracy of historical records	[10,11,44]
Mitochondrial DNA	Maternal ancestry, haplotypes and haplogroups	High maternal diversity; incomplete correspondence between mtDNA haplotypes and genealogical lines	Represents only the maternally inherited mitochondrial genome	[13,33,34]
Microsatellite markers	Nuclear diversity, heterozygosity, inbreeding and population differentiation	High allelic diversity, low inbreeding and weak differentiation among genealogical lines	Limited genomic resolution and dependence on the selected marker panel	[10,11]
Y-chromosome markers	Paternal ancestry and male haplotypes	Restricted paternal diversity; genealogical sire lines do not correspond to six distinct Y-chromosome lineages	Low polymorphism of the domestic horse Y chromosome	[13]
Whole-genome sequencing	Genome-wide population structure, ancestry and evolutionary relationships	Compact PKH genetic cluster, complex ancestry and no genomic separation matching founder lines	Current samples remain limited and unevenly distributed among lines	[14]

## Data Availability

The author declares that no new data were created.

## Abbreviation

The following abbreviation is used in this manuscript:

PKH	Polish Konik Horse
